# Development of Potentiometric Sensors for C_2_H_4_ Detection

**DOI:** 10.3390/s18092992

**Published:** 2018-09-07

**Authors:** Fidel Toldra-Reig, Jose M. Serra

**Affiliations:** Instituto de Tecnología Química, Universitat Politècnica de València—Consejo Superior de Investigaciones Científicas, Av. Los Naranjos s/n, E-46022 Valencia, Spain; fitolrei@itq.upv.es

**Keywords:** hydrocarbon, ethylene, potentiometric, sensor, YSZ, electrochemical cell

## Abstract

Gas exhaust emissions in vehicles are increasingly restrictive in EU and USA. Diesel engines are particularly affected by limitation in hydrocarbons and NOx concentrations. This work presents a screening of working electrode materials to develop a potentiometric sensor, with the most promising material to detect being C_2_H_4_ at 550 °C. The device consists of a dense 8YSZ (8 mol% Y_2_O_3_ stabilized ZrO_2_) disk as oxide-ion conducting electrolyte, whereas platinum is screen-printed in the back face as reference electrode. As working electrode, several materials such as Fe_0.7_Cr_1.3_O_3_, ZnCr_2_O_4_, Fe_2_NiO_4_, La_0.8_Sr_0.2_CrO_3−δ_ (LSC), La_0.8_Sr_0.2_MnO_3_ (LSM), and NiO+5%wt Au were tested to detect C_2_H_4_. Sensor voltage was measured for several concentrations of C_2_H_4_ and CO as these are two of the major oxidizable compounds in a diesel exhaust gas. Fe_0.7_Cr_1.3_O_3_ was selected as the most promising material because of its response to C_2_H_4_ and CO. Not only is the response to the individual analytes important, but the C_2_H_4_ cross-sensitivity toward CO is also important. Fe_0.7_Cr_1.3_O_3_ showed a good performance to C_2_H_4_, with low cross-sensitivity to CO. In addition, when 0.16 ppm of phenanthrene is added, the sensor still has a slightly better response to C_2_H_4_ than to CO. Nevertheless, the sensor exposure to high concentrations (>85 ppm) of polycyclic aromatic hydrocarbons led to signal saturation. On the other hand, the operation in wet conditions induces lower sensor sensitivity to C_2_H_4_ and higher cross-sensitivity toward CO increase, i.e., the sensor response becomes similar for C_2_H_4_ and CO.

## 1. Introduction

In the recent past years, the detection of hydrocarbons in exhaust gas of diesel cars has been increasing in importance because hydrocarbons, as well as other pollutants, are related to health issue problems in humans [1]. Then, both American and European legislators are reducing the permitted emissions limits of these pollutants [2,3,4]. Nowadays, there are restrictions because of the impossibility to detect HCs at lower concentrations. As a health issue, more restrictive legislations will be enforced if sensors able to detect HCs at this lower concentration level are technically and commercially available.

Potentiometric hydrocarbon sensors are the most appropriate candidates because of the high temperature of the exhaust gases and the gas composition and its simplicity and low cost. This type of sensor works as a solid-state electrochemical cell, where oxygen is reduced on the reference electrode and the oxygen ions generated diffuse through the solid electrolyte, e.g., 8YSZ (8 mol% Y_2_O_3_-stabilized ZrO_2_) and CGO (cerium gadolinium oxides), to oxidize the target gas (analyte) on the working electrode. Both electrodes—working and reference—are typically exposed to the same atmosphere, thus it is expected to behave as a mixed potential sensor. In fact, several reactions take place in each electrode and equilibrium is achieved. The reaction with the fastest kinetics rate will determine the electrochemical behavior of the sensor. On the other hand, the reference electrode could be exposed to another chamber where only oxygen in air is present.

Mixed potential sensors are preferred because both reference and working electrodes can be exposed to the same gas during operation. This makes the sensor suitable for exhaust automotive detection, e.g., detection of CO, NO_x_, HCs, NH_3_ [5,6,7]. In this sensor, the working electrode should have catalytic and sensing activity towards hydrocarbons, and low cross-sensitivity towards other gas components. On the other hand, the reference electrode should have catalytic activity toward oxygen reduction but must not convert other gas molecules (analytes). A proper selection of selective materials for the working and reference electrode is essential.

In the present work, several materials were screened to reach materials suitable for C_2_H_4_ detection with limited cross-sensitivity toward CO. The sensor device consists of a disk-shaped working solid 8YSZ membrane electrolyte [8,9,10] with a reference and a working electrode deposited on each face of the disk. Both electrodes are highly porous and were deposited by screen-printing. In literature, materials such as LaCoO_3_ [11], Nb_2_O_5_ [12], Au/8YSZ [8], SnO_2_ [13], Cr_2_O_3_ [13], La_2_CuO_4_ [14], TiO_2_(+Pd) [15], La_1−x_Sr_x_Cr_1−y_Ga_y_O_3−δ_ [16], Pt/YSZ, MgAl_2_O_4_ [17], and NiO/Au [18]. Thus, as working electrodes, several perovskites and metal oxides are studied in this work, similar to those aforementioned. The cell voltage is measured in atmospheres containing C_2_H_4_ and CO, as they are the most abundant partially oxidized compounds in diesel exhaust gas. A 6% fixed O_2_ concentration is employed, while the influence of water and aromatics is also studied. The most promising material is selected for a full characterization for ethylene detection purposes. One novel approach of the present work is the study of the cross-sensitivity: The sensor is exposed to a fixed concentration of one analyte while concentration pulses of a second analyte are performed. In literature, the cross-sensitivity is usually evaluated comparing the sensitivity of each element individually. Taking into account that competitive reactions can take place simultaneously in each electrode, single-gas measurements could not be adequate to evaluate the cross-sensitivity. Porous platinum is usually employed as reference electrode due to its high catalytic activity, thermal stability, and common use in sensor electrodes [19].

## 2. Materials and Methods

### 2.1. Synthesis of Electrode Materials

The starting precursors for the synthesis of working electrode materials were commercial nitrates from Sigma Aldrich. A sol-gel chemical route was followed to obtain single-phase perovskites and spinels. First, stoichiometric quantities of nitrate precursors were fully dissolved in water. After that, citric acid (Sigma Aldrich, St. Louis, MO, USA) was added as a chelating agent to prevent partial segregation or precipitation of the metal components, and then ethylene glycol was added to promote the polymerization of the chelating agent and produce an organometallic polymer resin (in a molar ratio 1:2:4 with respect to nitrates solution, citric acid, and ethylene glycol, respectively). This complexation step is followed by dehydration at low temperature (up to 200 °C) and, finally, thermal decomposition in air of the precursors at 600 °C led to the formation of nanosized crystalline phases.

The obtained powders were sintered at 1350 °C for 10 h and milled to produce the target crystalline phase and a homogenous particle size. Then, the material was mixed with an organic binder (terpineol with a 6%wt ethylcellulose) in a 1:2 weight ratio and passed through a three-roller mill to produce screen-printing inks. As reference electrode, a commercial platinum powder (particle size 0.2 to 1.8 µm, from Mateck (Jülich, Germany)) is employed and the same procedure is followed to obtain the ink. Several materials, such as Fe_0.7_Cr_1.3_O_3_, ZnCr_2_O_4_, Fe_2_NiO_4_, La_0.8_Sr_0.2_CrO_3−δ_ (LSC), La_0.8_Sr_0.2_MnO_3−δ_ (LSM), and NiO+5%wt Au, are employed as working electrodes. The aim was to find a material that provides a good response to ethylene. Once the most promising material was selected, further analyses were carried out with this configuration.

Only in the case of the Fe_0.7_Cr_1.3_O_3_, as the material alone did not attach properly to the 8YSZ electrolyte, was the powder was mixed with 8YSZ powder (Tosoh, Tokyo, Japan) to ensure a good electrode attachment. For this purpose, both powders in a 1:1 volume ratio were ball-milled for 24 h. Furthermore, this leads to an increase of the triple phase boundary length, i.e., the reaction sites for the electrochemical hydrocarbon oxidation.

### 2.2. Fabrication of the Sensor Device

**Figure 1:**
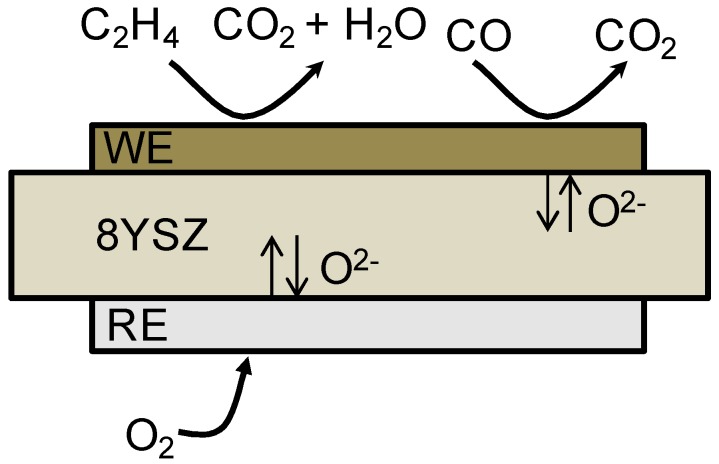
Scheme of the sensor. The device consists of a dense 8YSZ electrolyte with platinum reference electrode (**bottom**) and distinct materials as working electrode (**top**).

Individual 8YSZ disks of 20 mm of diameter were employed as electrolyte for the sensor assembly. The 8YSZ powder was uniaxially pressed at 50 kN into disks and then sintered at 1350 °C for 10 h. The device (Figure 1) is completed by screen-printing both circular electrodes (9 mm in diameter). First, the working electrode is printed and sintered at 1150 °C, and then platinum reference electrode is printed in the back face at the same conditions. Finally, a porous gold layer (mesh pattern) acting as current collector is screen-printed on top of the sintered working electrode, and sintered at 900 °C for 2 h. Gold is not expected to affect the sensor response as its catalytic activity depends critically on particle size. Bulk gold is chemically inert and is well known to be a very poor catalyst. It only has activity when employed as nanoparticle and at low temperatures [20,21]. Silver ink is employed to ensure contact between electrode and gold lead wires.

### 2.3. Characterization of Materials and Sensor Device

A PANalytical Cubix fast diffractometer, using CuK_α1_ radiation (*λ* = 1.5406 Å) and an X′Celerator detector in Bragg–Brentano geometry was used for the identification of the crystalline phases. XRD patterns recorded in the 2*θ*° range from 10° to 90° were analyzed using X’Pert Highscore Plus software (Malvern Panalytical, Malvern, UK). SEM and energy-dispersive X-ray spectroscopy (EDS) using a ZEISS Ultra55 field emission scanning electron microscope (ZEISS, Oberkochen, Germany) was used to analyze fracture cross-sections of the sintered material before and after the permeation test. SEM backscattered electrons detector (BSD) was used to provide images with compositional contrast that differentiate grains and element distribution.

The voltage was determined by measuring the potential difference between the working and reference electrode when a zero-ampere current is applied. Gold wires and silver ink were used for contacting electrodes. The measurements were carried out at 550 °C and several concentrations of C_2_H_4_ and/or CO were measured (Figure S1). The cell voltage between both electrodes was detected by a multimeter, Keithley 3706 (Keithley Instrument, Cleveland, OH, USA). The response of the sensor (V_cell_, mV) was defined as:

V_cell_ = V_a_ − V_b_ (1)

where V_a_ and V_b_ are the voltage of the sensor exposed to (a) analytes with the background gas of 6% O_2_ (in Ar balance), and (b) background gas alone (6% O_2_ in Ar balance), respectively.

Mass flow controllers were used to obtain different gas mixtures. The total gas flow rate was 550 mL/min with a 6% of oxygen, varying C_2_H_4_ and CO concentrations and Ar as balance. Once the sample is stabilized at 550 °C, C_2_H_4_ or CO concentration was varied from 50 ppm (used as base gas) to 100, 150, and 200 ppm. The same test was performed for each gas with 200 ppm of the other gas as background in order to check cross-sensitivity. The same tests are performed with a background of water (3%) and polyaromatics: Toluene (28,947 ppm), methylnaphtalene (88 ppm), and phenanthrene (0.16 ppm). The gas flow is saturated in these elements at room temperature and then the selection of polyaromatics is limited to (1) their vapor pressure, and (2) the fact that polyaromatics concentration in exhaust gases is usually in the range from 0 to 10 ppm [22,23,24]. The aromatics are selected according to its vapor pressure to provide a concentration close to the desired range: One compound with a much higher concentration than expected in an exhaust gas (toluene), as it could be interesting to check the performance under extreme conditions, and two compounds closer to the range in exhaust gases (methylnaphthalene and phenanthrene).

Electrochemical impedance spectroscopy analysis was carried for each analyte at 200 ppm using an Autolab PGSTAT204 (Metrohm Autolab, Utrecht, The Netherlands) with a FRA32M module. The frequency was changed from 0.03 Hz to 1 MHz. Prior to the EIS measurement, open circuit voltage (OCV) is measured and this bias voltage is applied for the EIS measurement. The polarization resistance is measured as the difference between the high frequency and low frequency intercepts with the real axis of the impedance (Z’).

Coke formation on the surface of the electrode was studied by Raman spectroscopy. Raman spectrometer is equipped with a Leica DMLM microscope (Leica Microsystems, Wetzlar, Germany) and a 785-nm Ar^+^ ion laser as an excitation source. A 50× objective of 8-mm optical length was used to focus the depolarized laser beam on a spot of 3 μm in diameter. A charged coupled device (CCD) array detector was used for the Raman scattering collection.

## 3. Results and Discussion

### 3.1. Microstructural Characterization

**Figure 2:**
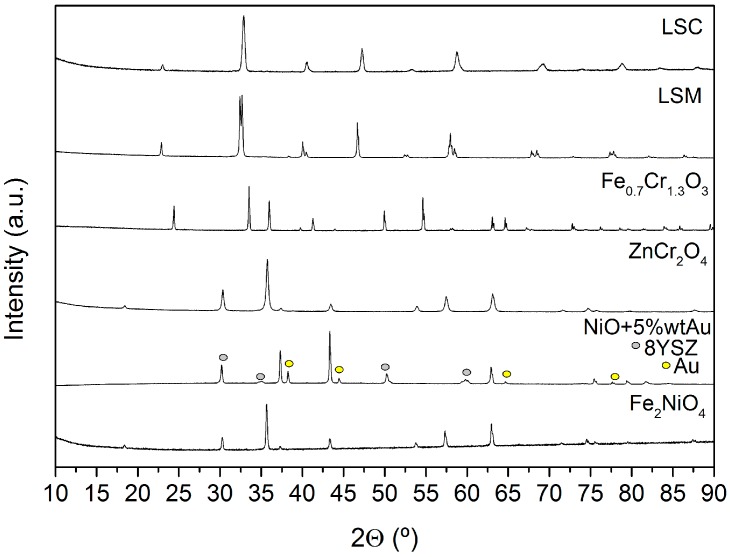
X-ray diffraction patterns of the materials studied at room temperature once they are synthesized.

Analysis of the X-ray diffraction (XRD) patterns (Figure 2) of the different as-sintered electrode powders confirms that the target crystalline phase was obtained for each material. Diffraction peaks corresponding to secondary phases are not observed. 

**Figure 3:**
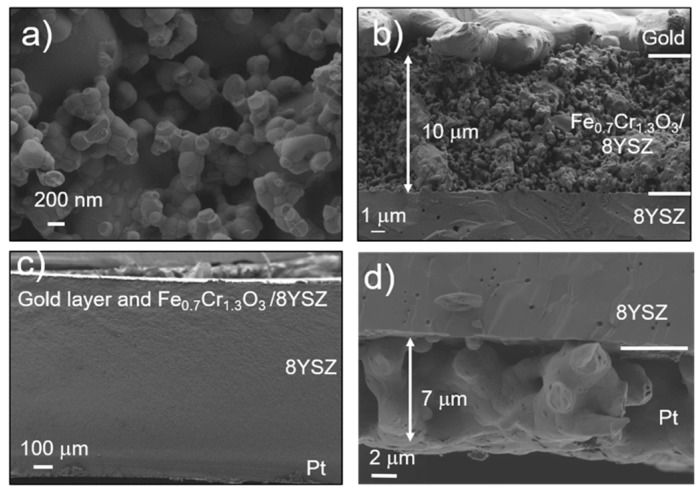
SEM image of the device cross-section corresponding to FeCr_2_O_4_/8YSZ as WE and Pt as RE. (**a**) WE electrode cross-section with FeCr_2_O_4_ and 8YSZ grains. (**b**) Interface WE-electrolyte where a good attachment of the layer can be observed. Gold collector layer at the top as well as FeCr_2_O_4_ and 8YSZ grain distribution can be observed. (**c**) Complete device Fe_0.7_Cr_1.3_O_3_/8YSZ || 8YSZ || Pt can be observed. (**d**) Sintered platinum reference can be observed.

SEM analysis (Figure 3 and Figure S2 in Supporting Information) shows the structural and morphologic characteristics of the built sensor device. The fracture cross-section of the porous working electrode (Figure 3a,b) reveals a distinct grain size distribution for Fe_0.7_Cr_1.3_O_3_ and 8YSZ, i.e., Fe_0.7_Cr_1.3_O_3_ grains are bigger. Nevertheless, a homogeneous distribution of grains is reached throughout the 10 µm-thick electrode. No reaction interfaces are detected between electrodes and electrolyte. Neither reaction between the phases nor impurities on the grain boundaries are detected (Figure 3b–d). The reference electrode is a 7 µm-platinum layer made of highly-coarsened grains with good adhesion to the electrolyte. Figure 3c shows a view of the complete device with a ~1mm-thick dense 8YSZ electrolyte. For all electrodes with different compositions, a proper attachment of electrodes to the 8YSZ substrate was achieved, excepting La_0.8_Sr_0.2_CrO_3−δ_ (LSC) electrode that was detached.

### 3.2. Electrochemical Characterization

In the presence of CO, hydrocarbons, other reducing agents, and oxygen, several oxidation and reduction reactions take place at the interface among electrode, electrolyte, and gas phase (mixed potential sensor). In a potentiometric sensor, a zero-current is applied. Thus, competitive reactions take place in both electrode and therefore, kinetics will be a key point. When a steady-state between the anodic reaction (Equation (3) or Equation (4)) and the cathodic reaction Equation (2) is achieved, a mixed-potential is established. The difference between the mixed-potential established in both electrodes will give rise to the voltage of the cell [25]. Then, the sensor performance can be enhanced with a proper selection of the materials: Selective material to the desired target analyte in the WE, and a material active to oxygen as RE (Figure 1). Note that both electrodes are exposed to the same gas mixture, i.e., RE is not exposed to a reference gas (air). In addition, heterogeneous catalytic conversion in the WE of the target gases (analytes) with locally adsorbed O_2_ can occur at the same time Equations (5) and (6). This reaction will compete with the (sensing) electrochemical reactions and affect the sensor response if the electrode is not active towards electrochemical oxidation of C_2_H_4_. These are the specific reactions taken into account [26].

(2) $$\;\frac{1}{2}O_{2}+2e^{-}\to O^{2-}\;$$

(3) $$\;\mathrm{CO}+O^{2-}\to {\mathrm{CO}}_{2}+2e^{-}\;$$

(4) $$\;C_{2}H_{4}+6O^{2-}\to 2{\mathrm{CO}}_{2}+2H_{2}O+12e^{-}\;$$

(5) $$\;C_{2}H_{4}+3O_{2}\to 2{\mathrm{CO}}_{2}+2H_{2}O\;$$

(6) $$\;\mathrm{CO}+0.5O_{2}\to {\mathrm{CO}}_{2}\;$$

In a first set of experiments, several perovskites, spinels, and other oxides were studied employing platinum as reference electrode. The aim is to identify promising materials for hydrocarbon sensing applications in an environment such as diesel exhaust. Thus, a high response to C_2_H_4_ and a low response to CO are required. Sensors were exposed to several concentrations of each gas. In a first screening, the sensors were exposed to a concentration range from 200 to 1000 ppm and, in a second step, the sensors were exposed to a concentration range from 50 to 200 ppm.

**Figure 4:**
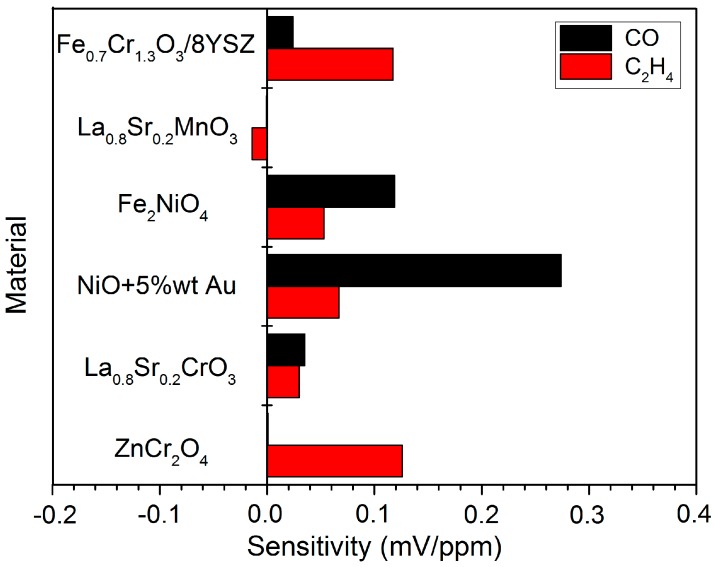
Sensor sensitivity (mV/ppm) to C_2_H_4_ (red) and CO (black) individually for several materials used as working electrode. The sensor sensitivity is defined as V_cell_/analyte concentration (mV/ppm) in the linear range of sensor response and calculated as a linear regression. ZnCr_2_O_4_ and Fe_2_NiO_4_ are evaluated in the range 200–1000 ppm, while the rest of the materials (Fe_0.7_Cr_1.3_O_3_, LSC, LSM, and NiO+5%wt Au) are evaluated in the range 50–200 ppm.

As the sensor responses are linear with the analyte concentration within the studied range, the sensor sensitivity (Figure 4) is defined as the slope dV_cell_/dC_analyte_ (mV/ppm) and calculated as a linear regression. NiO+5%wt Au, FeNiO_4_, and NiO show higher response to CO rather than C_2_H_4_, and were further investigated. LSC and LSM show low response with low sensitivity toward C_2_H_4_. ZnCr_2_O_4_ and Fe_0.7_Cr_1.3_O_3_ exhibit promising C_2_H_4_ sensitivity, i.e., high response to C_2_H_4_ and low response to CO. Nevertheless, ZnCr_2_O_4_ response is not stable with time despite providing the highest response to C_2_H_4_. Thus, the sensor response is not reproducible and not appropriate for long-term operation. This could be due to an ageing effect, e.g., sintering and coking (Figure S3 in Supporting Information). Thus, Fe_0.7_Cr_1.3_O_3_ is a priori the most promising sensing material because of the stable response and appropriate response to C_2_H_4_.

The mixture of Fe_0.7_Cr_1.3_O_3_ with 8YSZ ensures good attachment to the electrolyte and enables to enlarge the number of active sites available for the electrochemical hydrocarbon oxidation, i.e., the triple phase boundary (TPB) length. Therefore, apart from the intrinsic catalytic activity of the electrode material, the sensor performance can be boosted because of the increment of the TPB area. Then, in order to disclose if the good performance of Fe_0.7_Cr_1.3_O_3_ is due to the catalytic activity of the material itself or because of the increment of the TPB area, another tested material (LSC) is mixed with 8YSZ (1:1 volume ratio) and exposed to C_2_H_4_. When compared to the bare LSC electrode, no significant effect is observed (Figure S4) and therefore the good performance of the Fe_0.7_Cr_1.3_O_3_-8YSZ can be attributed to the intrinsic Fe_0.7_Cr_1.3_O_3_ activity.

**Figure 5:**
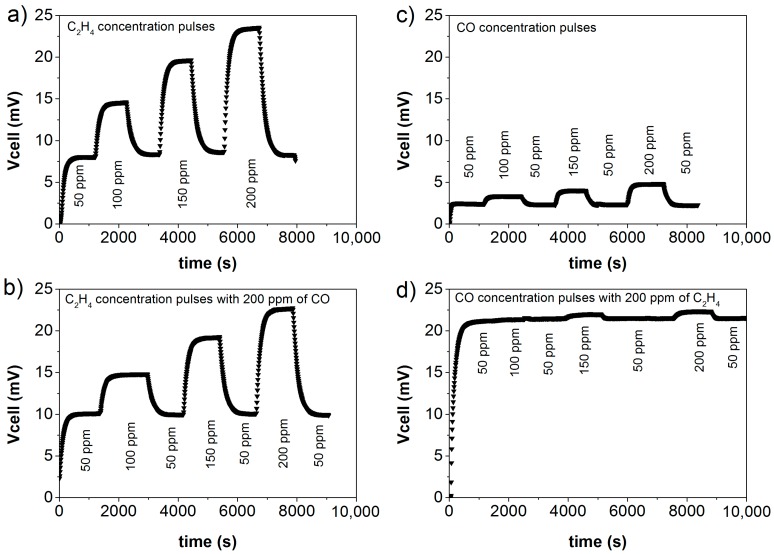
Response curves for changes in concentration of: (**a**) C_2_H_4_, (**b**) C_2_H_4_ with 200 ppm CO background, (**c**) CO, and (**d**) CO with 200 ppm C_2_H_4_ background. Each change in concentration has 20 min duration and goes from 50 ppm to 100, 150, and 200 ppm. The sensor consists of Fe_0.7_Cr_1.3_O_3_/8YSZ as WE and Pt as RE, at 550 °C with 6% of O_2_.

Sensors based on 8YSZ-Fe_0.7_Cr_1.3_O_3_ working electrode were further characterized by exposing them to C_2_H_4_ and CO mixtures with several concentrations. Specifically, a background concentration (200 ppm) of one analyte is set when varying the concentration of the other analyte. Figure 5 shows the recorded V_cell_ as a function of time for series of stepwise changes in gas composition, and illustrates high response sensitivity to C_2_H_4_ (Figure 5a), together with low cross-sensitivity toward CO (Figure 5b). Indeed, the sensor hardly responds to changes in CO concentration when C_2_H_4_ (at 200 ppm level) is present in the gas. Further, this figure reveals that the reproducibility and stability of the signal upon equilibration, while response kinetics is rather fast. Additionally, the sensor baseline is close to zero and stable without any drift observed (Figure S5).

**Figure 6:**
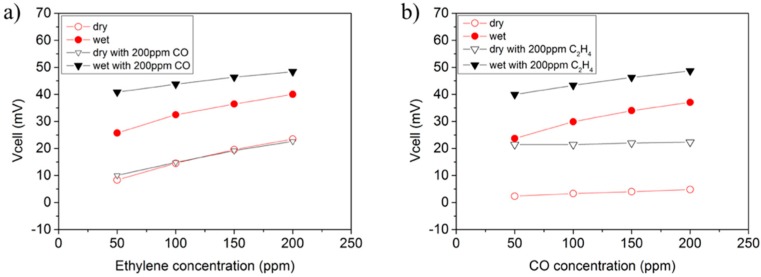
EMF Sensor response as a function of analyte concentration for: (**a**) C_2_H_4_ and (**b**) CO. The effect of a background concentration (200 ppm) of the second analyte and 3% of water is also depicted. Each point is the main of the respective 20 min change in response.

Figure 6 plots the equilibrium V_cell_ as a function of CO/C_2_H_4_ concentration. In dry conditions (open symbols in Figure 6), the sensor response (V_cell_) is proportional to C_2_H_4_ concentration regardless of the CO background, whereas the response to CO changes is nearly negligible. The same study was repeated by adding 3% of water (saturation of inlet gases at room temperature). The addition of water (solid symbols in Figure 6) strongly increases the sensitivity of CO while slightly affects sensitivity to C_2_H_4_ and, consequently, leads to higher cross-sensitivity to CO (Table 1). Two effects may be responsible for this behavior: (1) Water could promote CO conversion by removing surface coke from surface or avoiding its formation; and (2) water enables the formation of H_2_ on the surface of both WE and RE (Pt) via water gas-shift reaction (WGS, Equation (7)). The electrochemical oxidation of the locally-built H_2_ on the electrodes is kinetically more favored than CO oxidation and this may have a notable impact in the sensor response to CO.

(7) $$\;H_{2}O+\mathrm{CO}\to {\mathrm{CO}}_{2}+H_{2}\;$$

**Figure 7:**
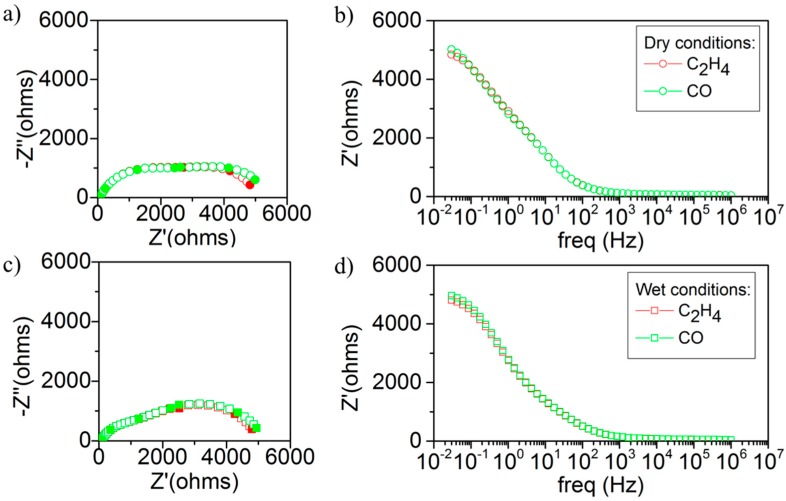
Impedance spectrometry study in dry and wet conditions for Ar+6%O_2_, 200 ppm C_2_H_4_, and 200 ppm CO. (**a**) Nyquist plot and (**b**) Bode plot in dry conditions, and (**c**) Nyquist plot and (**d**) Bode plot in wet conditions with a 3% of H_2_O.

Figure 7 displays the electrochemical impedance spectra (Nyquist and Bode plots, frequency range 0.03 Hz to 1 MHz) of the sensor exposed to 200 ppm of C_2_H_4_ or CO, both in dry and wet conditions. Electrochemical impedance spectroscopy (EIS) analysis provides information related with the processes taking place in the device (ionic transport, solid-solid interfacial resistance, gas-solid surface reactions) since they may exhibit distinct characteristic frequencies. For the present case, the variation of the analyte concentration or type may induce changes in the impedance contributions only related to gas-solid surface reactions and appearing at the lowest frequencies. The larger the impedance, the slower (or less favored) the specific surface reaction (oxidation of analytes in this case) is. Although the results are rather similar for both analytes, there is a slight difference at low frequencies for both analytes (impedance is slight lower in C_2_H_4_) that can be linked to the results observed in the potentiometric characterization. In dry conditions (Figure 7a,b), the shape of the arc at high frequencies is similar for both analytes, therefore the ionic transport remains almost unaffected. On the other hand, the response impedance (Z’) at low frequencies is sensitive to changes in the gas composition and this suggests that the sensor response depends on electrochemical reactions at the surface of Fe_0.7_Cr_1.3_O_3_/8YSZ and interface with 8YSZ electrolyte [27,28,29,30,31]. Specifically, the low frequency response is higher in CO than in C_2_H_4_ (Table 2) and is in line with the observed sensor behavior in dry conditions. In wet conditions (Figure 7c,d), the impedance arc at low frequency is practically unaltered in C_2_H_4_ but it is reduced in CO (Table 2). Water has an effect on the electrochemical reactions taking place in both the electrode and electrode-electrolyte interface, as observed previously in the potentiometric characterization. Thus, the electrochemical CO evolution is ameliorated on the electrode surface thanks to the presence of water, likely mediated by locally formed H_2_ (Equation (7)). Therefore, the decrease in polarization resistance (Rp) results in higher sensitivity to CO and therefore in loss of the desired sensor performance to C_2_H_4_.

**Figure 8:**
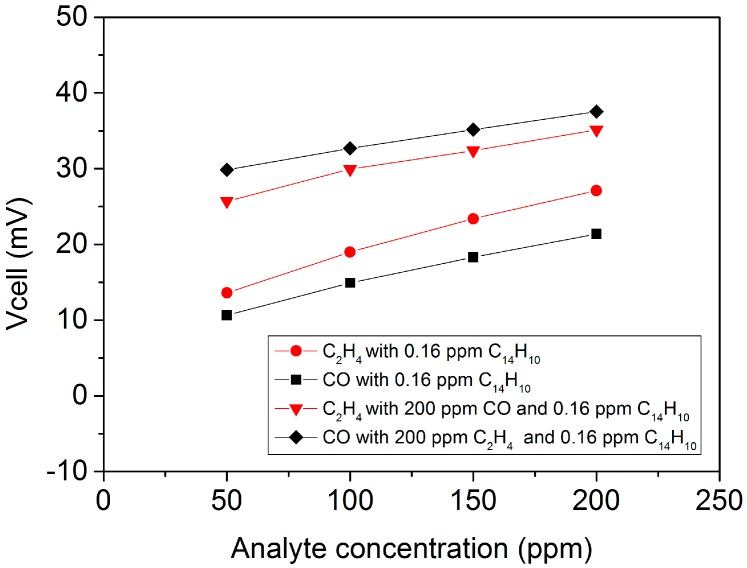
Sensor response when 0.16 ppm of phenantrene is added as a function of analyte concentration, C_2_H_4_ in red, and CO in black. The effect of background concentration (200 ppm) of the second analyte is depicted. Each point is the average of the respective 20 min change in response.

As a final test of the sensor based on 8YSZ-Fe_0.7_Cr_1.3_O_3_ working electrode, the effect of the exposure to minor amounts of different aromatic hydrocarbons on the potentiometric response was studied in dry conditions (Table 3), as they are potential candidates in exhaust gases that can interfere in the sensor response to C_2_H_4_. For this purpose, the gas feed was separately saturated at room temperature with toluene (28,947 ppm), methylnaphtalene (88 ppm), and phenanthrene (C_14_H_10_-0.16 ppm) at room temperature. The sensor shows high sensitivity to C_14_H_10_ while the response is still slightly higher in C_2_H_4_ than in CO (Table 1). However, the difference in response becomes small when one analyte is evaluated in the presence of 200 ppm of the second analyte (Figure 8 and Table 1), i.e., the sensor response to C_2_H_4_ is practically lost in presence of C_14_H_10_. Similarly, toluene addition leads to high cross-sensitivity toward CO and C_2_H_4_, while toluene response signal is superimposed over both CO and C_2_H_4_ responses, although it should be taken into consideration that toluene and methylnaphtalene concentration is out of the range of exhaust gases. Additionally, the sensitivity of the sensor toward poly-aromatics seems to increase with the number of rings. This could be related to the higher reactivity of larger aromatics.

Regarding sensor aging upon test in aromatics, a single exposure to one of these aromatics gives rise to changes in response when newly measured without aromatics, specifically the sensitivity decays. If a regeneration step is done (sensor calcination at 600 °C with air), C_2_H_4_ and CO response recover the original behavior. This suggests that coke may be formed on the surface of the electrodes. The iron spinel oxide provides Lewis acidic sites that have a catalytic activity toward aromatics oxidation and oligomerization [32]. Iron oxide is more affected by aging and coke formation leading to a reduction of its catalytic activity [33]. Thus, coke effect should be removed and probably this could be due to a surface modification when aromatics are partly oxidized during sensor operation. The sensor was exposed to working conditions at 550 °C for 24 days and negligible ageing effect was noticed on the sensor response.

**Figure 9:**
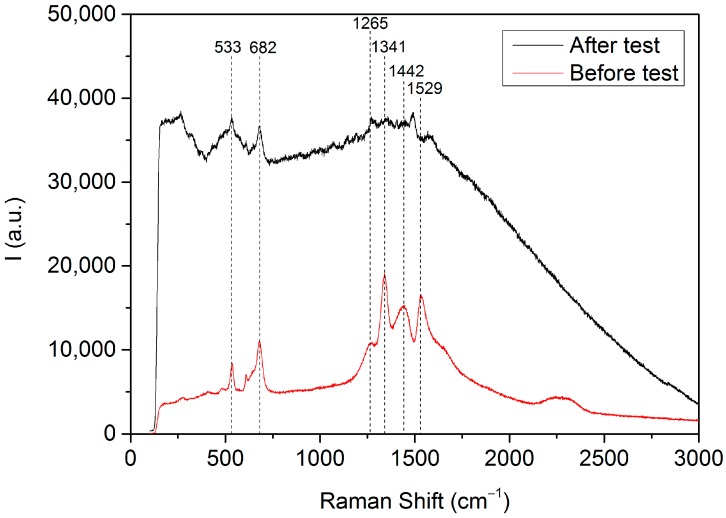
Raman spectra of the Fe_0.7_Cr_1.3_O_4_/8YSZ electrode layer with a gold collector after and before test.

Thus, as coke formation is suspected to happen after exposure to aromatics, Raman spectroscopy analyses were performed over the Fe_0.7_Cr_1.3_O_3_/8YSZ electrode layer with a gold collector before and after the test in order to check the coke formation. Raman spectroscopy is a technique that enables one to identify the potential presence of surface species related to distinct carbon forms. Figure 9 depicts both Raman spectra where the peaks corresponding to the spinel phase can be noticed at band values of 533 and 682 cm^−1^ [34,35,36]. Another common peak corresponding to Fe_3_O_4_ can be detected before and after at 1341 cm^−1^ [36]. The Raman spectra of the tested sample present more peaks in the region from 1200 to 1600 cm^−1^ than the untested sample. Amorphous carbon (1340–1400 cm^−1^ and 1540–1600 cm^−1^ [37]) and carbon nanotubes (1562 cm^−1^ [38]) are expected to appear in this region. In addition, there is much more fluorescence when the tested sample is measured. This may indicate the presence of complex aromatic molecules adsorbed on the electrode surface and this is in agreement with the lack of sensitivity after exposure to the aforementioned aromatics (and its recovery after a regeneration step). Despite these considerations, carbon fingerprint is here not detected to the limit of this technique.

Regarding NO_2_ cross sensitivity on the RE, Pt is well known for its catalytic activity toward O_2_ at mid-high temperatures. Although NO_2_ is a common compound in diesel exhaust gas, according to other potentiometric sensors for selective NO_2_ detection, the catalytic activity of Pt to oxygen redox reaction is expected to be much higher than the NO_2_ reduction reaction [7,39,40,41]. Thus, there should not be a significant effect on the sensor response, although experimental assays are required.

## 4. Conclusions

Fe_0.7_Cr_1.3_O_3_ was identified as a suitable working electrode material for C_2_H_4_ detection in exhaust gas streams that contain oxidizable gases together with molecular oxygen. The tested sensor cell comprised a disk-shaped solid oxide-ion conducting electrolyte (8YSZ) coated with the working electrode and a reference electrode (Pt) on the other side. This cell assembly Fe_0.7_Cr_1.3_O_3_/8YSZ || 8YSZ || Pt provides an adequate response to C_2_H_4_, with low cross-sensitivity to CO at 550 °C. This makes the sensor suitable for sensing in conditions like exhaust gases from diesel cars. The study using wet gas streams or adding significant concentration of polycyclic aromatic hydrocarbons (PAHs) revealed the need of further adjusting of surface properties of electrodes to keep high response towards C_2_H_4_ and signal stability, regardless of the presence of variable amounts of water or PAHs in ppm level. In the case of water, the activity toward water gases-shift reaction should be minimized in both working and reference electrodes.

## Figures and Tables

**Table 1:** Sensitivity of the sensor to C_2_H_4_, carbon monoxide, and each analyte with 200 ppm of background of the second analyte for dry and wet (3%) conditions, as well as for 0.16 ppm of C_14_H_10_. Sensor sensitivity is defined as V_cell_/analyte concentration (mV/ppm) and calculated as a linear regression.

Analyte	Dry Sensitivity (mV/ppm)	Wet Sensitivity (mV/ppm)	C_14_H_10_ Sensitivity (mV/ppm)
C_2_H_4_	1.2 × 10^−1^	9.4 × 10^−2^	9.0 × 10^−2^
CO	2.4 × 10^−2^	8.8 × 10^−2^	7.1 × 10^−2^
C_2_H_4_ with 200 ppm of CO	8.4 × 10^−2^	5.1 × 10^−2^	6.1 × 10^−2^
CO with 200 ppm of C_2_H_4_	6.6 × 10^−3^	5.8 × 10^−2^	5.1 × 10^−2^

**Table 2:** Polarization resistance (Rp) as a function of analyte (C_2_H_4_ and carbon monoxide) in dry and wet conditions. In both conditions the concentration of the analytes are 200 ppm.

	Analyte (200 ppm)	Rp (ohm)
dry	C_2_H_4_	5112.52
	CO	5548.20
wet	C_2_H_4_	5079.02
	CO	5257.03

**Table 3:** Sensor response to several conditions: Ar+6%O_2_ base gas, toluene, methylnaphtalene, phenantrene, C_2_H_4_, and carbon monoxide. Concentration, cell voltage, and sensor sensitivity as mV/ppm are provided.

Compound	Cell Voltage (mV)	Concentration (ppm)	Sensitivity (mV/ppm)
Ar+6%O_2_	0.03	-	-
C_7_H_8_	119.16	28,947.37	0.004
C_11_H_10_	−158.75	88.16	−1.80
C_14_H_10_	0.23	0.16	1.44
C_2_H_4_	23.49	200	0.12
CO	4.82	200	0.02

## Supplementary Materials

The following are available online at http://www.mdpi.com/1424-8220/18/9/2992/s1, Figure S1: Experimental set-up used to measure sensor response, Figure S2: SEM image of the device cross-section showing the interface WE-electrolyte corresponding to (a) FeNiO_4_, (b) ZnCr_2_O_4_, (c) LSM and (d) LSC. On the other hand, (e) shows the NiO+5%wt Au surface, Figure S3: Response curves for changes in concentration of ZnCr_2_O_4_ with fresh ink and 1 month ink: (a) response to 400 ppm C_2_H_4_ with fresh paste, (b) response to changes in concentration from 50 to 200 ppm of C_2_H_4_. The sensor consists of ZnCr_2_O_4_ as WE and Pt as RE and at 550 °C with a 6% of O_2_ and balanced with Ar. A lower sensor response can be observed after 1 month of paste storage. Thus, the material reproducibility does not fit the requirements about stability and reproducibility with time, Figure S4: Response transient to C_2_H_4_ of the sensor employing as WE: (a) LSC and (b) LSC/8YSZ (1:1 vol.), Figure S5: Sensor baseline when exposed to 6% of O_2_ balanced with argon at 550 °C. The sensor consists of Fe_0.7_Cr_1.3_O_3_/8YSZ as WE and Pt as RE.
