# Supplementary material for: Development of Potentiometric Sensors for C2H4 Detection
Source: Sensors (Basel). 2018 Sep 7;18(9):2992. doi: 10.3390/s18092992 (PMC6164524; doi:10.3390/s18092992)
Supplement: Supplementary file 1 [file sensors-18-02992-s001.pdf]

Supporting information

Development of potentiometric sensors to C_2H_4

F. Toldra-Reig, J.M. Serra

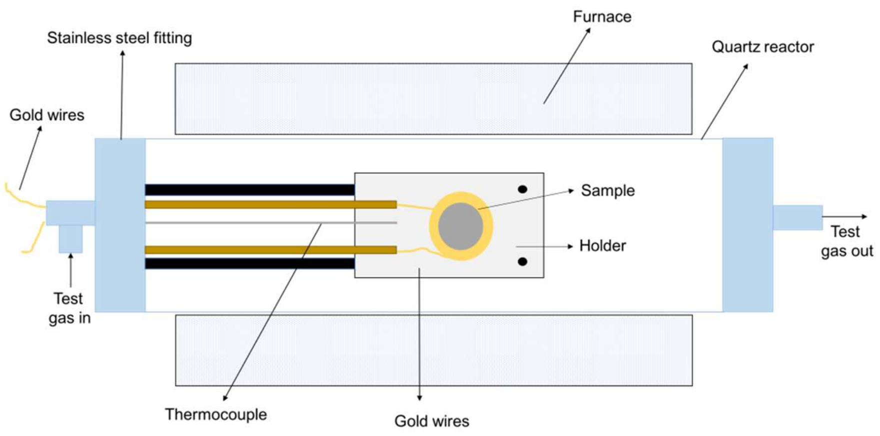

Figure S1 Experimental set-up used to measure sensor response

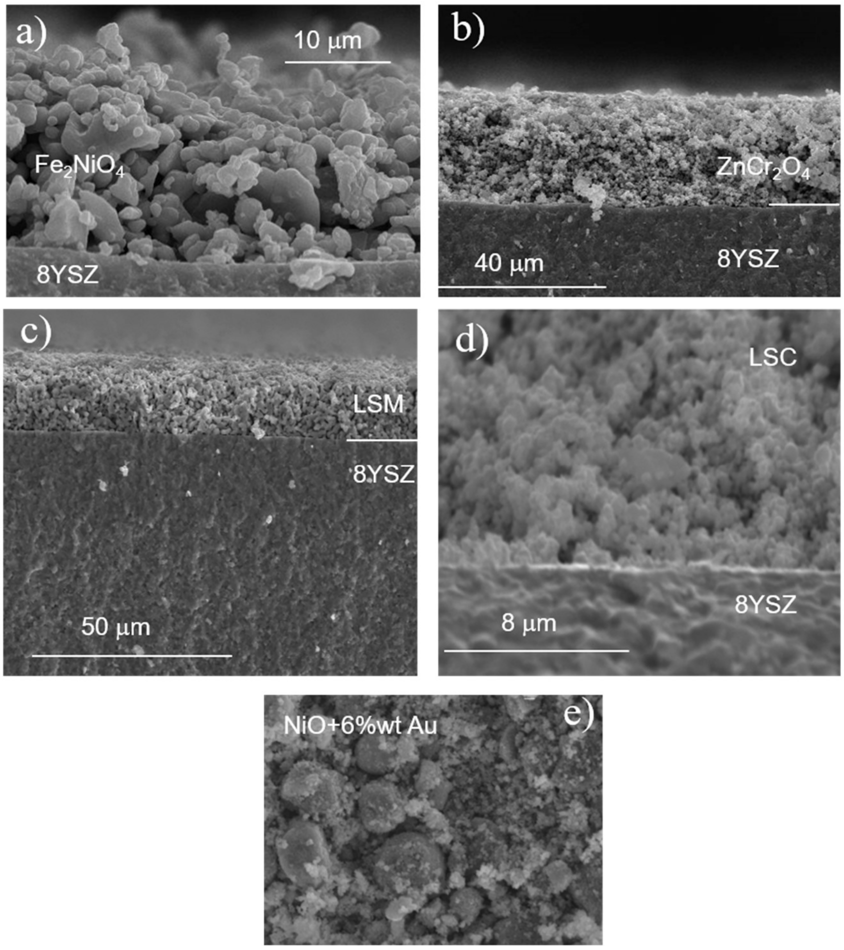

Figure S2 SEM image of the device cross-section showing the interface WE-electrolyte corresponding to a) $FeNiO_4$, b) $ZnCr_2O_4$, c) LSM and d) LSC. On the other hand, e) shows the NiO+5%wt Au surface.

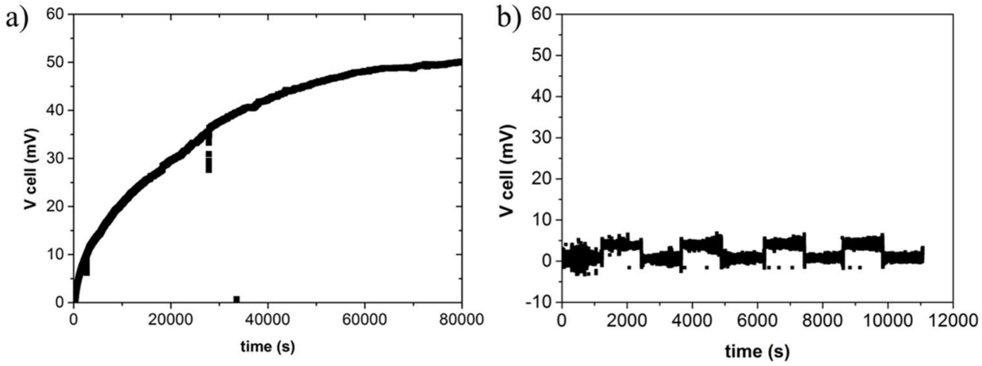

Figure S3 Response curves for changes in concentration of $ZnCr_2O_4$ with fresh ink and 1 month ink: a) response to 400 ppm C_2H_4 with fresh paste, b) response to changes in concentration from 50 to 200 ppm of C_2H_4. The sensor consists of $ZnCr_2O_4$ as WE and Pt as RE and at 550ºC with a 6% of O_2 and balanced with Ar. A lower sensor response can be observed after 1 month of paste storage. Thus, the material reproducibility does not fit the requirements about stability and reproducibility with time.

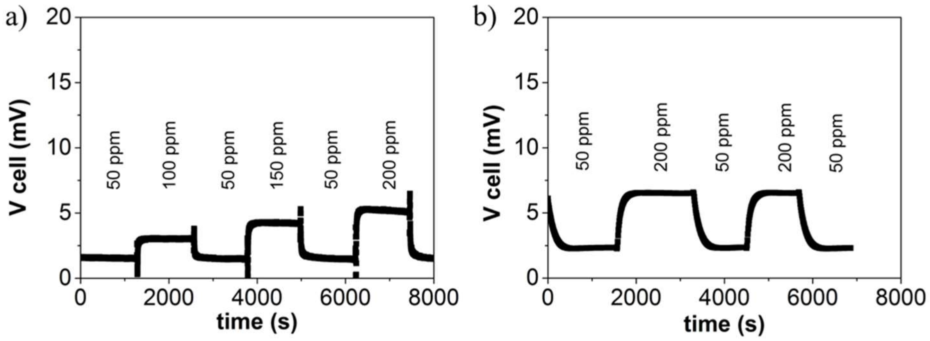

Figure S4 Response transient to C_2H_4 of the sensor employing as WE: a) LSC and b) LSC/8YSZ (1:1 vol.)

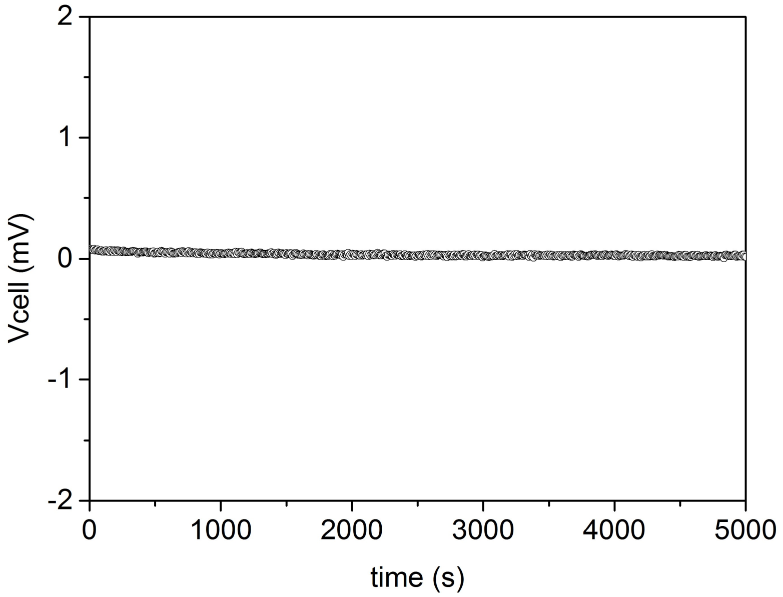

Figure S5 Sensor baseline when exposed to 6% of O_2 balanced with argon at 550 ºC. The sensor consists of $Fe_{0.7}Cr_{1.3}O_3$/8YSZ as WE and Pt as RE
